# Increased financial burdens and lengths of stay in patients with healthcare-associated infections due to multidrug-resistant bacteria in intensive care units: A propensity-matched case-control study

**DOI:** 10.1371/journal.pone.0233265

**Published:** 2020-05-18

**Authors:** Li-Hsiang Su, I-Ling Chen, Ya-Fen Tang, Jen-Sin Lee, Jien-Wei Liu

**Affiliations:** 1 Infection Control Team, Kaohsiung Chang Gung Memorial Hospital, Kaohsiung, Taiwan; 2 Department of Pharmacy, Kaohsiung Chang Gung Memorial Hospital, Kaohsiung, Taiwan; 3 School of Pharmacy, Kaohsiung Medical University, Kaohsiung, Taiwan; 4 Faculty of Finance, I-Shou University, Kaohsiung, Taiwan; 5 Division of Infectious Diseases, Department of Internal Medicine, Kaohsiung Chang Gung Memorial Hospital, Kaohsiung, Taiwan; 6 Chang Gung University Medical College, Taoyuan, Taiwan; Nazareth Hospital, UNITED STATES

## Abstract

**Background and objectives:**

Incidence rates of healthcare-associated infections (HAIs) depend upon infection control policy and practices, and the effectiveness of the implementation of antibiotic stewardship. Amongst intensive care unit (ICU) patients with HAIs, a substantial number of pathogens were reported to be multidrug-resistant bacteria (MDRB). However, impacts of ICU HAIs due to MDRB (MDRB-HAIs) remain understudied. Our study aimed to evaluate the negative impacts of MRDB-HAIs versus HAIs due to non-MDRB (non-MRDB-HAIs).

**Methods:**

Among 60,317 adult patients admitted at ICUs of a 2680-bed medical centre in Taiwan between January 2010 and December 2017, 279 pairs of propensity-score matched MRDB-HAI and non-MRDB-HAI were analyzed.

**Principal findings:**

Between the MDRB-HAI group and the non-MDRB-HAI group, significant differences were found in overall hospital costs, costs of medical and nursing services, medication, and rooms/beds, and in ICU length-of-stay (LOS). As compared with the non-MDRB-HAI group, the mean of the overall hospital costs of patients in the MDRB-HAI group was increased by 26%; for categorized expenditures, the mean of costs of medical and nursing services of patients in the MDRB-HAI group was increased by 8%, of medication by 26.9%, of rooms/beds by 10.3%. The mean ICU LOS in the MDRB-HAI group was increased by 13%. Mortality rates in both groups did not significantly differ.

**Conclusions:**

These data clearly demonstrate more negative impacts of MDRB-HAIs in ICUs. The quantified financial burdens will be helpful for hospital/government policymakers in allocating resources to mitigate MDRB-HAIs in ICUs; in case of need for clarification/verification of the medico-economic burdens of MDRB-HAIs in different healthcare systems, this study provides a model to facilitate the evaluations.

## Introduction

The vulnerabilities of patients admitted to intensive care units (ICUs) make them subject to high risk for healthcare-associated infections (HAIs), and therapeutically-indicated invasive procedures/devices add to the problem [1, 2]. ICU-associated HAIs contribute to increased mortality, financial expenditures, and ICU length-of-stay (LOS) [3]. When it comes to etiologies, HAIs acquired from ICU setting are subject to remarkably high risk of being caused by multidrug-resistant bacteria (MDRB) [4, 5]. Incidence of HAIs due to MDRB (MDRB-HAIs) was reported to be as high as 40% in many world regions [5]. However, impacts of ICU HAIs due to MDRB (MDRB-HAIs) have not been fully addressed. Incidence rates of HAIs depend on infection control policy and practices [6–12], and the effectiveness of the implementation of antibiotic stewardship [4, 8, 13–16]. The emergence of MDRB has long been found to result from the selective pressure of extensive antibiotic exposures [13–15], and antibiotic stewardship is therefore especially important in diminishing the incidence of MDRB-HAIs [4, 13–18]. With the hypothesized more profound negative impacts of MDRB-HAIs as compared with those produced by HAIs due to non-MDRB (non-MRDB-HAIs), the objective of this study was to evaluate impacts of MRDB-HAIs versus non-MRDB-HAIs in ICUs in a large medical centre in Taiwan. Clarifications of the impacts of MRDB-HAIs will help government/hospital policymakers plan on how to effectively allocate resources to mitigate the problems.

## Patients and methods

### Study design, patients, hospital setting and antibiotic stewardship

This is retrospective case-control study comparing the differences in financial burdens, hospital/ICU LOS and mortality in adult patients with MRDB-HAIs and those with non-MDRB-HAIs admitted at ICUs of Kaohsiung Chang Gung Memorial Hospital (KSCGMH), a 2680-bed facility serving as a primary care and tertiary referral medical centre in southern Taiwan. There are 12 adult ICUs in KSCGMH, which were categorized as ICU(s) of general medicine (n = 4), general surgery (n = 2), neurology (n = 2), neurosurgery (n = 2), cardiology (n = 1), and thoracic cardiovascular surgery (n = 1). Patients aged ≧ 18 years admitted to one of these ICUs between January 2010 and December 2017 suffering either a MRDB-HAI or non-MDRB-HAI were included for propensity-score matching (PSM) for evaluation. Participants were fully anonymized before their data being accessed, and the Institutional Review Board of Chang Gung Memorial Hospital waived the requirement of participants’ informed consent (Document no. 201800460B0C501). The study would be reported in adherence with the recommended principle [19].

HAIs were defined as infections that were not present and lacking evidence of incubation at the time of admission to an ICU [20]. HAIs and the pathogens were identified based on the CDC diagnostic criteria for nosocomial infections at the hospital’s regular surveillance [20]. With the exception of surgical site infections, no specific time during or after hospitalization was given to determine whether or not an infection was healthcare-associated; each infection was assessed for evidence linking to acquisition at hospitalization [20]. A surgical site infection was regarded as healthcare-associated in case it occurred within 30 days after surgery if no implant was left in place or within 1 year if an implant was in place [20].

KSCGMH’s regular HAI surveillance was carried out by the staff composed of the same experienced infection-control practitioners throughout the study period under the supervision of a senior infectious-diseases specialist (Dr. J-W Liu) [8]. Along with other hospitals in Taiwan, the HAI incidence density (HAI episodes per 1000 intensive care unit-days [‰]) of KSCGMH was reported on a quarterly-basis to Taiwan CDC for regular nationwide HAI surveillance [21, 22]. The HAI incidence densities at adult ICUs of KSCGMH were 9.2‰ in 2010 and 5.4‰ in 2017; with the exception of a slightly elevated HAI incidence density of 6.7‰ found in 2016, a progressively decreasing trend in HAI incidence density was observed during the study period (S1 Fig).

The implementation of antibiotic stewardship in KSCGMH is briefly described as follows [8]. All antimicrobial prescriptions at any ICU had to be made online, which were then subjected to review by a dedicated infectious-diseases specialist. The reviewer was able to access online the clinical, laboratory, radiographic information of the patient in question for evaluation before making an approval or disapproval decision. The decision and the reviewer’s comment/suggestion were immediately texted to the antimicrobial prescribers and the pharmacy. Prescribed antimicrobial(s) was immediately dispensed, and would be continuously dispensed by the pharmacy only when the prescription was approved online. In case of disapproval, the antimicrobial(s) would be discontinued 48 hours after being prescribed.

### Term definitions

MDRB referred to any of the following bacterial isolates: methicillin-resistant *Staphylococcus aureus* (MRSA), vancomycin-resistant *Enterococcus* (VRE), extended spectrum β-lactamases (ESBLs)-producing *Entertobacteriaceae*, carbapenem-resistant *Entertobacteriaceae*, multidrug-resistant (MDR)-*Pseudomonas aeruginosa*, MDR-*Acinetobacter* spp., and MDR-*Stenotrophomonas maltophilia*. The identifications of the pathogens for HAIs and the susceptibility testing were performed on clinical-practice basis, as were described elsewhere [8, 23]. MDR-*P*. *aeruginosa* and MDR-*Acinetobacter* spp. were defined based on the criteria proposed by Magiorakos, et al. with modifications [8, 24]. Specifically, a MDR-*P*. *aeruginosa* was regarded as a *P*. *aeruginosa* isolate that was resistant to ≧one agent in three or more of the antibiotic classes included in the tested antibiotic profile for non-glucose-fermenting Gram-negative bacilli, which included aminoglycosides, anti-pseudomonal carbapenems, anti-pseudomonal cephalosporins, anti-pseudomonal fluoroquinolones, piperacilliln/tazobactam, and polymyxins. A MDR-*Acinetobacter* species referred to an *Acinetobacter* isolate that, in addition to being resistant to ≧ one agent in three or more of the antibiotic classes in the tested antibiotic profile for non-glucose-fermenting Gram-negative bacilli, was resistant to extended-spectrum cephalosporins, folate pathway inhibitors, ampicillin/sulbactam, polymyxins and tigecycline. MDR-*S*. *maltophilia* referred to a *S*. *maltophilia* isolate that was resistant to folate pathway inhibitors and levofloxacin/moxifloxacin.

Mortality referred to all-cause death during each patient’s hospital stay. Financial burdens referred to the overall hospital costs, which were, for further analyses, categorized into costs of medical and nursing services, medication, diagnostic and laboratory tests, ancillary services (i.e., pharmacy, radiology and physical therapy services), rooms/beds, and others. Assessments of financial burdens in New Taiwan dollars (NT$) were carried out using the database retrieved from the inpatient hospital costs submitted by KSCGMH’s administrative department to National Healthcare Insurance, a single-payer compulsory public healthcare insurance system, started in 1995 and has been covering nearly 100% of the population in Taiwan [25]. The list of patients stayed at ICUs was retrieved from this database as well. Comparisons of the financial burdens, mortality rates, hospital LOS, and ICU LOS between patients with MDRB-HAI and those with non-MDRB-HAI were performed. Increased medical expenditures and increased hospital/ICU LOS for MDRB-HAIs were respectively defined as the differences in medical expenditures and in hospital/ICU LOS between the MDRB-HAI and the non-MDRB-HAI groups.

### Statistical analysis

A logistic regression model was constructed to calculate propensity scores which were the probability of assignment conditional on the observed baseline characteristics of both the MDRB-HAI and the non-MDRB-HAI groups [26–28]. Covariates included in the regression model were gender, age, underlying diseases, hospital LOS before admission to an ICU, Acute Physiologic Assessment and Chronic Health Evaluation II (APACHE II) scores [29], Charlson comorbidity index [30], and individual ICUs (see Table 1 for details).

**Table 1:** Demographic and clinical characteristics of patients with HAIs in the pre-propensity-score matched and propensity-score matched cohorts and comparisons between patients with MDRB-HAIs and those with non-MDRB-HAIs.

Variable	Overall patients (n = 1003)	Pre-propensity-score matched			Propensity-score matched		
		Patients with MDRB-HAI (n = 378)	Patients with non-MDRB-HAI (n = 625)	*P*^a^	Patients with MDRO-HAI (n = 279)	Patients with non-MDRO-HAI (n = 279)	*P*^a^
Age, year (mean ± SD)	66.2 ± 15.3	68.2±15.0	64.9 ±15.3	<0.001	67.8±15.3	68.4 ± 14.2	0.866
Gender				0.639			0.861
Male	609 (60.7)	226 (59.8)	383 (61.3)		176 (63.1)	174 (62.4)	
Female	394 (39.3)	152 (40.2)	242 (38.7)		103 (36.9)	105 (37.6)	
Diabetes mellitus				0.027			0.988
Without end organ damage	185 (28.4)	123 (32.5)	162 (25.9)		89 (31.9)	89 (31.9)	
With end organ damage^b^	84 (8.4)	36 (9.5)	48 (7.7)		24 (8.6)	25 (9.0)	
Myocardial infarction	159 (15.9)	63 (16.7)	96 (15.4)	0.583	48 (17.2)	48 (17.2)	1
Congestive heart failure	238 (23.7)	117 (31.0)	121 (19.4)	<0.001	81 (29.0)	84 (30.1)	0.780
Connective tissue disease	29 (2.9)	10 (2.6)	19 (3.0)	0.171	8 (2.9)	10 (3.6)	0.631
Liver disease				0.054			0.647
Mild liver disease^c^	193 (19.2)	76 (20.1)	117 (18.7)		58 (20.8)	56 (20.1)	
Moderate to severe liver disease^d^	49 (4.9)	26 (6.9)	23 (3.7)		20 (7.2)	15 (5.4)	
Peripheral vascular disease or bypass	83 (8.3)	26 (6.9)	57 (9.1)	0.211	22 (7.9)	18 (6.5)	0.511
COPD/asthma	227 (22.6)	110 (29.1)	117 (18.7)	0.001	76 (27.2)	75 (26.9)	0.924
LOS in hospital prior to admission to ICU, day (mean ± SD)	2.40 ± 6.48	2.6 ± 7.5	2.2 ± 5.7	0.093	2.1 ± 6.0	2.2 ± 6.4	0.808
APACHE II score (mean ± SD)	21.31 ± 8.48	22.9 ± 8.6	20.3 ± 8.1	0.133	22.8 ± 8.7	22.8 ± 8.3	0.953
Charlson index score (mean ± SD) [10]	3.91 ± 3.03	4.3 ± 3.1	3.6 ± 2.9	<0.001	4.1 ± 2.9	4.0 ± 2.9	0.665
Categorized ICU				<0.001			0.992
General medicine	247 (24.6)	128 (33.9)	119 (19.0)		92 (33.0)	91 (32.6)	
General, surgery	140 (14.0)	46 (12.2)	94 (15.0)		28 (10.0)	27 (9.7)	
Neurology	245 (24.4)	90 (23.8)	155 (24.8)		74 (26.5)	76 (27.2)	
Neurosurgery	182 (18.1)	51 (13.5)	131 (21.0)			40 (14.3)		38 (13.6)	
Cardiovascular Surgery	91 (9.1)	27 (7.1)	64 (10.2)			17 (6.1)		15 (5.4)	
Cardiology	98 (9.8)	36 (9.5)	62 (9.9)	28 (10.0)		32 (11.5)			

Figures in the table referred to number of patients (%), unless stated otherwise.

HAIs, healthcare-associated infections; MDRO-HAIs, healthcare-associated infections due to multidrug-resistant bacteria; non-MDRO-HAIs, healthcare-associated infections due to non-multidrug-resistant bacteria; SD, standard deviation; COPD, chronic obstructive pulmonary disease; ICU, intensive care unit; APACHE II score, Acute Physiologic Assessment and Chronic Health Evaluation II [9]; LOS, length of stay

^a^ For comparisons between patients with MDRB-HAI and patients with non-MDRB-HAI

^b^ Including retinopathy, neuropathy and/or nephropathy

^c^Referring to chronic hepatitis or liver cirrhosis without portal hypertension

^d^Referring to liver cirrhosis and portal hypertension, without or with variceal bleeding history.

The PSM was performed for patients of the MDRB-HAI group and those of the non-MDRO-HAI group at a 1:1 ratio, without replacement, by the nearest neighbor technique, as was prescribed elsewhere [26–28]. The propensity-score matched MDRB-HAI and non-MDRB-HAI groups were compared with each other in terms of mortality rate, medical expenditures, hospital LOS and ICU LOS.

For hypothesis testing between different groups, the Student’s *t*-test was used for normally distributed continuous variables, and the Mann-Whitney *U* test for skewed distributions; the χ^2^ test or Fisher’s exact test was used for categorical variables, as necessary [31]. Differences were considered statistically significant at a *P* < 0.05. Data retrieval and statistical analyses were performed using the SAS software package, version 9.0 (SAS Institute Inc., NC).

## Results

**Fig 1:**
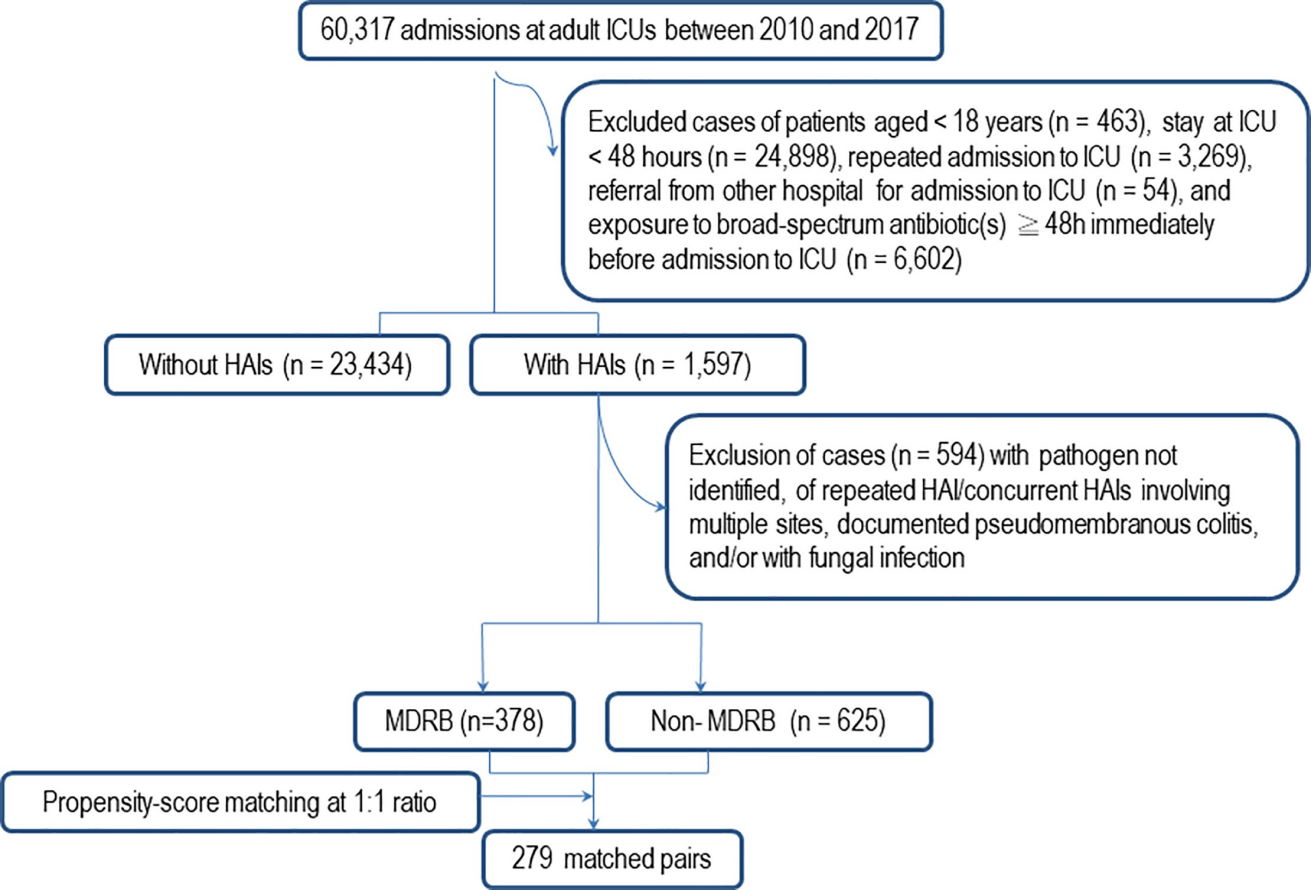
Flow chart of selection and exclusion of healthcare-associated infections due to multidrug-resistant bacteria (MDRB-HAIs) and those due to non-multidrug-resistant bacteria (non-MDRB-HAIs) at intensive care units (ICUs), and propensity-score matching.

Among the overall 60,317 admissions at adult ICUs in KSCGMH during the study period, 1597 adults suffered HAIs, while 23,434 did not, indicating that 6.4% of patients acquired HAI(s) during their stay at an ICU (Fig 1); of note, 1,003 cases (378 [37.7%] MDRB-HAIs and 625 [62.3%] non-MDRB-HAIs) each involved one patient with one episode of HAI were included as eligibility for potential PSM. Eventually, 279 pairs each composed of one case from the MDRB-HAI group and another from the non-MDRB-HAI group were propensity-score matched. Flow chart for detailed case selection and exclusion for PSM is shown in Fig 1. The included patients were elderly and male predominant, with multiple comorbidities and advanced clinical severity. Characteristics of the overall included patients and patient allocations based on MDRB-HAIs and non-MDRB-HAIs before and after PSM are shown in Table 1. The propensity-score matched groups were well balanced.

The 5 leading pathogens in the MDRB-HAI group were *E*. *coli* (n = 112), methicillin-resistant *S*. *aureus* (n = 35), *Klebsiella pneumoniae* (n = 26), *A*. *baumannii* (n = 24), VRE (n = 21), while those in the non-MDRB group were *P*. *aeruginosa* (n = 82), *Enterococcus* spp. (n = 43), *E*. *coli* (n = 35), coagulase-negative staphylococci (n = 20), and *S*. *maltophilia* (n = 9) (S2 Fig). Major annually found HAI entities at ICUs included urinary tract infection/blood stream infection, followed by pneumonia or surgical site infection (S3 Fig).

Between the MDRB-HAI group and the non-MDRB-HAI group, significant differences were found in the overall hospital costs, costs of medical and nursing services, medication, and rooms/beds, and in ICU LOS. As compared with the counterpart figures in the non-MDRB-HAI group, the mean of the overall hospital costs of patients in the MDRB-HAI group was increased by 26%; for categorized expenditures, the mean of costs of medical and nursing services was increased by 8%, of medication by 26.9%, and of rooms/beds by 10.3%. The mean ICU LOS was increased by 13% (Table 2). Between the MDRB-HAI and non-MDRB-HAI groups, mortality rates and hospital LOS did not significantly differ.

**Table 2:** Comparisons of the overall hospital costs and costs of detailed items, hospital LOS, ICU LOS, and mortality rate between patients with MDRB-HAIs and with non-MDRB-HAIs.

Variable	MDRB-HAIs (A)	Non-MDRB-HAIs (B)	*P* value^a^	Difference (%)^b^
Over hospital costs, mean ($NT)	532,311.2 ± 315,333.5	474,264.0 ± 298,839.5	**0.013**	16,622.2 (26)
Medical and nursing services	25,368.7 ± 11,904.4	23,499.2 ± 11,278.8	**0.027**	1,869.5 (8.0)
Medication	82,867.1 ± 102,111.5	65,322.8 ± 75,268.4	**<0.001**	17,544.3 (26.9)
Rooms/beds	174,290.3 ± 87975.9	158,051.8 ± 87405.7	**0.012**	16,238.5 (10.3)
Diagnostic laboratory tests	44,363.9 ± 37582.8	40,586.1 ± 29047.3	0.118	3777.8 (9.3)
Ancillary services^c^	156,391.6 ± 132405.8	140,373.5 ± 114236.6	0.094	16018.1 (11.4)
Others	49,029.6 ± 95775.5	46,430.6 ± 93223.3	0.382	2599 (5.6)
Hospital LOS, mean (days)	50.1 ± 33.9	49.1 ± 35.9	0.431	1.0 (2)
ICU LOS, mean (days)	25.2 ± 13.8	22.4 ± 13.8	**0.006**	2.8 (13)
Mortality rate, %	110 (39.4)	95 (34.0)	0.188	6 (6.3%)

MDRB-HAIs, healthcare-associated infections due to multidrug-resistant bacteria; non-MDRB-HAIs, healthcare-associated infections due to non-multidrug-resistant bacteria; LOS, length of stay.

^a^For comparison between A and B.

^b^%: Derived from A-B/B × 100.

^c^Support services other than medical and nursing services, as well as room and board provided to hospitalized patients; they include pharmacy, radiology and physical therapy services.

*P* value marked in bold font indicates statistical significance.

## Discussion

The emergence of MDRB in hospital settings has been involving a large number of microbe species. Among them, *E*. *faecium*, *S*. *aureus*, *K*. *pneumoniae*, *A*. *baumanii*, *P*. *aeruginosa*, and *Enterobacter* species, which were collectively dubbed “ESKAPE”, have been found to be major pathogens for the majority of HAIs and to effectively “escape” the effects of antibiotics [32, 33]. Previously published researches on HAI impacts were based on the analyses of HAIs caused by the individual ESKAPE members of the same species but differing in antimicrobial susceptibility, and disclosed that MDRB-HAIs linking to a specific ESKAPE member led to significantly higher financial burdens and patient mortality rates [34]. However, MDRB emerged in hospital settings are not limited to ESKAPE members [33].

Based on the PSM with balanced measured baseline covariates of the comparators [26], and rather than assessing the impacts of MDRB-HAIs caused by a specific ESKAPE member, we evaluated the impacts of MDRB-HAIs caused by a wide variety of pathogens including all ESKAPE members and other bacterial species. Our evaluation results are more truly reflective of the overall financial burdens and clinical outcomes of MDRB-HAIs in the real-world clinical setting of ICUs. To minimize confounding, however, at PSM we excluded patients with exposure to broad-spectrum antibiotic(s) for more than 48 hours immediately prior to admission to an ICU (see Fig 1), and some clinically severe patients might therefore be precluded from being analyzed. This was likely to result in biases in assessing clinical outcomes and financial burdens of MDRB-HAIs in ICUs.

Of note, 6.4% of patients acquired HAI(s) during their stay in an ICU in KSCGMH at the study period, which was comparable to the HAI prevalence ranged from 4.6% to 9.3% in EU hospitals [35]. Prevalence of HAIs greatly varies among hospitals in different geographic locales, and is associated with a large number of factors such as prolonged and inappropriate use of invasive devices and antibiotics, high-risk and sophisticated procedures, patients’ comorbidities and immunocompromised conditions, poor knowledge and application of basic infection control measures, insufficient application of standard and isolation precautions, inadequate environmental hygienic conditions and waste disposal, suboptimal hospital infrastructure, insufficient equipment, lack of local and national guidelines and policies, understaffing, and overcrowding [36].

HAI in ICUs has been a well-documented independent risk factor for mortality [1]. Similar mortality rates found in different patient groups in our PSM cohort suggested a similar vulnerability of patients admitted at our ICUs, regardless of whether they suffered a non-MDRB-HAI or MDRB-HAI. Similar mortality rates and the overall hospital LOS in the non-MDRB-HAI and MDRB-HAI groups were reflective of the comparable advanced clinical severity and the multiple comorbidities in patients in both groups, as were indicated by the high APACHE II scores and Charlson index scores. Despite similar mortality rates in the non-MDRB-HAI and MDRB-HAI groups, the significantly increased ICU LOS in the latter, as was observed in other reports [37], may account for the increase in healthcare demand, which in turn led to the increase in financial burdens in the MDRB-HAI group.

Of note, *Candida* species were not included as multidrug-resistant organisms in this study because susceptibility testing for fungi has not been carried out on clinical- practice basis in most hospitals including ours, despite the fungi being isolated from normally sterile sites. In addition, standards for antimicrobial susceptibility testing are not well established for fungi, not to mention a consensus on defining multidrug-resistance in fungal species [38]. Candidemia has been one of the leading HAIs [38, 39], and prolonged hospitalization and extensive antibiotic exposure have been the documented 2, alongside other well-known risk factors for acquisition of invasive *Candida* infections [39, 40]. It should be regarded as a limitation to this study in which all *Candida* infections were universally excluded from HAIs due to multidrug-resistant organisms, while the susceptibilities of the pathogenic *Candida* species were not determined.

## Conclusions

Our data, estimated based on MDRB-HAIs vs. non-MDRB-HAIs, highlighted the more profound negative impacts of MDRB-HAIs on financial burdens and ICU LOS. This report quantified the medico-economic burdens of MDRB-HAIs in ICUs, which will be helpful for hospital/government policymakers in planning how to allocate resources to mitigate the incidence of MDRB-HAIs in ICUs, and this is especially important in healthcare practice with global-budget-based medical reimbursements. In case of need for clarification/verification of the medico-economic burdens of MDRB-HAIs in different healthcare systems, our report provides a model to facilitate the evaluations.

## Supporting information

S1 Fig Healthcare-associated infection incidence densities at adult intensive care units of Kaohsiung Chang Gung Memorial Hospital between 2010 and 2017. (TIF)

S2 Fig Multidrug-resistant bacteria (MDRB) (n = 279) and non-MDRB (n = 184) are shown. The rest 95 non-MDRB (no.) included *Enterococcus faecalis* (19), coagulase-negative stphylococci (34), *Serratia maltophilia* (9), *Pseudomonas mirabilis* (7), *Achromobacter xylosoxidans* (4), *Elizabethkingia meningoseptica* (3), *Morganella morganii* (3), Group-B *Streptococcus* (2), *Citrobacter diversus* (2), and *Acinetobacter lwoffii*, *Aeromonas hydrophila*, *Comamonas acidovorans*, *Corynebacterium* sp., *Enterococcus* sp., *Haemophilus influenzae*, *Pseudomonas* sp., *Salmonella enterica* serogroup D, *Streptococcus constellatus*, *Streptococcus pneumoniae*, *Staphylococcus saprophyticus*, viridans streptococcus each one isolate.
(TIF)

S3 Fig Healthcare-associated infection entities at adult intensive care units of Kaohsiung Chang Gung Memorial Hospital. BSI, bloodstream infection; UTI, urinary tract infection; SSI, surgical site infection; PNEU, pneumonia.
(TIF)
