# Supplementary figures and images for: Increased financial burdens and lengths of stay in patients with healthcare-associated infections due to multidrug-resistant bacteria in intensive care units: A propensity-matched case-control study
Source: PLoS One. 2020 May 18;15(5):e0233265. doi: 10.1371/journal.pone.0233265 (PMC7233534; doi:10.1371/journal.pone.0233265)

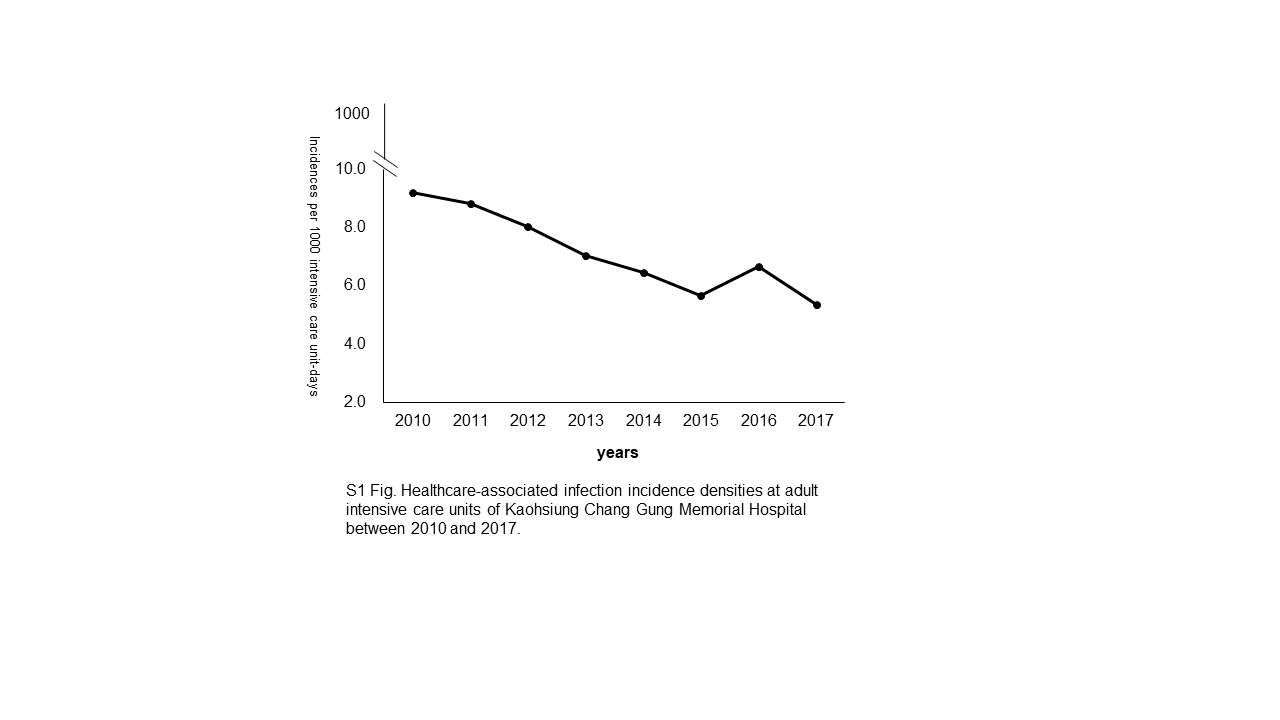

Supplement: S1 Fig — (TIF) [file pone.0233265.s001.tif]

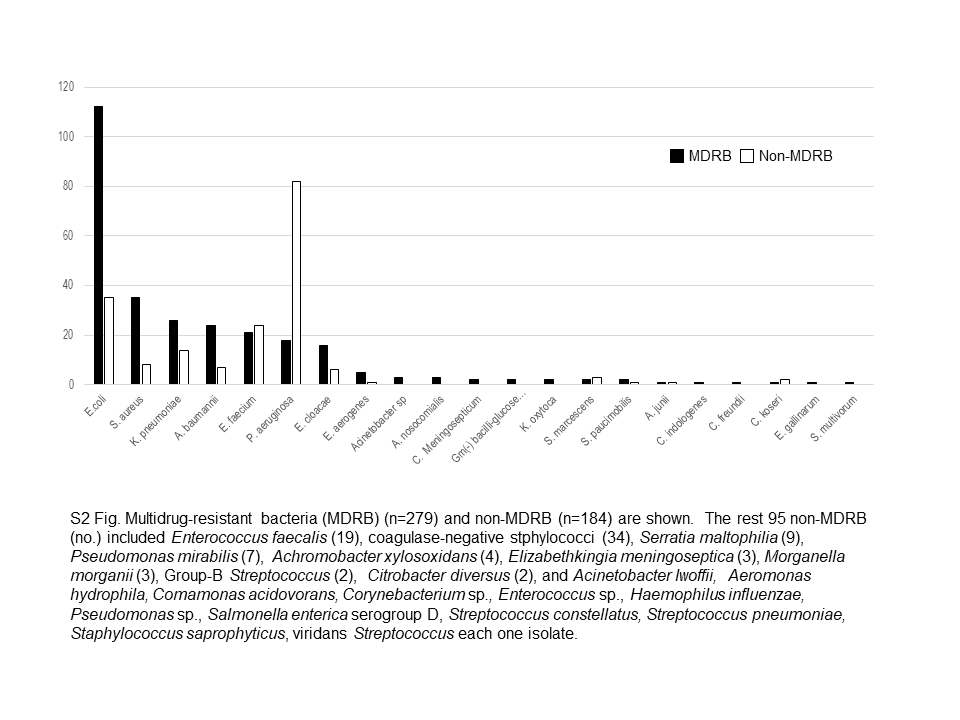

Supplement: S2 Fig — The rest 95 non-MDRB (no.) included Enterococcus faecalis (19), coagulase-negative stphylococci (34), Serratia maltophilia (9), Pseudomonas mirabilis (7), Achromobacter xylosoxidans (4), Elizabethkingia meningoseptica (3), Morganella morganii (3), Group-B Streptococcus (2), Citrobacter diversus (2), and Acinetobacter lwoffii, Aeromonas hydrophila, Comamonas acidovorans, Corynebacterium sp., Enterococcus sp., Haemophilus influenzae, Pseudomonas sp., Salmonella enterica serogroup D, Streptococcus constellatus, Streptococcus pneumoniae, Staphylococcus saprophyticus, viridans streptococcus each one isolate. (TIF) [file pone.0233265.s002.tif]

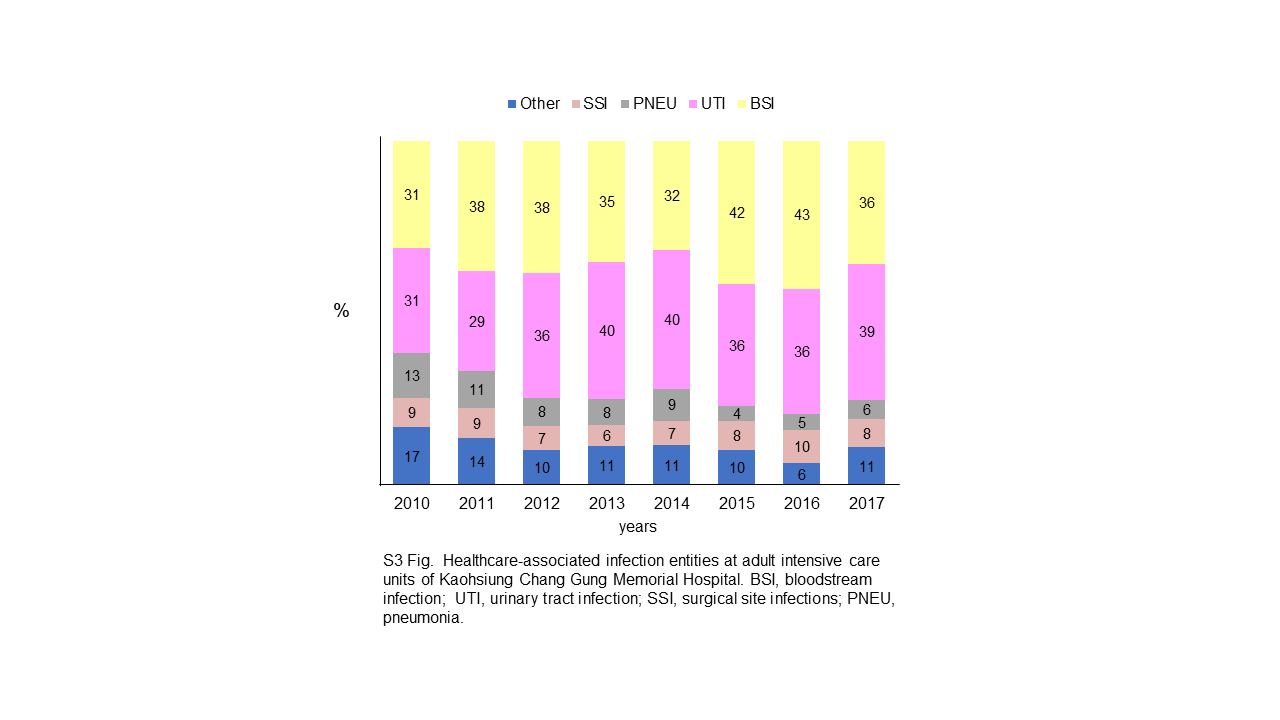

Supplement: S3 Fig — BSI, bloodstream infection; UTI, urinary tract infection; SSI, surgical site infection; PNEU, pneumonia. (TIF) [file pone.0233265.s003.tif]
